# Early transcriptome changes associated with western diet induced NASH in *Ldlr*^−/−^ mice points to activation of hepatic macrophages and an acute phase response

**DOI:** 10.3389/fnut.2023.1147602

**Published:** 2023-08-07

**Authors:** Jyothi Padiadpu, Melinda H. Spooner, Zhipeng Li, Nolan Newman, Christiane V. Löhr, K. Denise Apperson, Amiran Dzutsev, Giorgio Trinchieri, Natalia Shulzhenko, Andrey Morgun, Donald B. Jump

**Affiliations:** ^1^College of Pharmacy, Oregon State University, Corvallis, OR, United States; ^2^Nutrition Program, Colleges of Public Health and Human Sciences, Oregon State University, Corvallis, OR, United States; ^3^College of Veterinary Medicine, Oregon State University, Corvallis, OR, United States; ^4^Cancer and Inflammation Program, Center for Cancer Research, National Cancer Institute, National Institutes of Health (NCI-NIH), Bethesda, MD, United States

**Keywords:** NAFLD, NASH, steatosis, fibrosis, metabolic syndrome, hepatic transcriptome, NASH associated macrophage, biological network

## Abstract

**Background:**

Nonalcoholic fatty liver disease (NAFLD) is a global health problem. Identifying early gene indicators contributing to the onset and progression of NAFLD has the potential to develop novel targets for early therapeutic intervention. We report on the early and late transcriptomic signatures of western diet (WD)-induced nonalcoholic steatohepatitis (NASH) in female and male *Ldlr*^−/−^ mice, with time-points at 1 week and 40 weeks on the WD. Control *Ldlr*^−/−^ mice were maintained on a low-fat diet (LFD) for 1 and 40 weeks.

**Methods:**

The approach included quantitation of anthropometric and hepatic histology markers of disease as well as the hepatic transcriptome.

**Results:**

Only mice fed the WD for 40 weeks revealed evidence of NASH, i.e., hepatic steatosis and fibrosis. RNASeq transcriptome analysis, however, revealed multiple cell-specific changes in gene expression after 1 week that persisted to 40 weeks on the WD. These early markers of disease include induction of acute phase response (*Saa1-2, Orm2*), fibrosis (*Col1A1, Col1A2, TGFβ*) and NASH associated macrophage (NAM, i.e., *Trem2 high, Mmp12 low*). We also noted the induction of transcripts associated with metabolic syndrome, including *Mmp12, Trem2, Gpnmb, Lgals3* and *Lpl*. Finally, 1 week of WD feeding was sufficient to significantly induce TNFα, a cytokine involved in both hepatic and systemic inflammation.

**Conclusion:**

This study revealed early onset changes in the hepatic transcriptome that develop well before any anthropometric or histological evidence of NALFD or NASH and pointed to cell-specific targeting for the prevention of disease progression.

## Introduction

Nonalcoholic fatty liver disease (NAFLD) is the most common form of chronic fatty liver disease worldwide; ~25% of the global population is estimated to have some level of NAFLD (1–3). The World Health Organization reported over 1.9 billion overweight adults in 2016; and this level of obesity parallels the global increase in NAFLD patients (1, 2, 4, 5). The National Health and Nutrition Examination Survey (NHANES) estimates nearly 40% of adults in the US are obese (2). Obesity severity increases the incidence of NAFLD ranging from 75% in overweight individuals to over 90% in morbidly obese individuals (1, 2, 6, 7). NAFLD is associated with metabolic syndrome (MetS) and MetS is linked to obesity, type 2 diabetes mellitus (T2DM), dyslipidemia and hypertension. These are the top four risk factors associated with NAFLD, and they are strongly associated with patients with a BMI > 30 (1, 4).

From a clinical perspective, NAFLD is a continuum of fatty liver diseases ranging from simple steatosis to nonalcoholic steatohepatitis (NASH, the progressive form of disease), cirrhosis, hepatocellular carcinoma (HCC) and liver failure (8, 9). NAFLD occurs in children and adults, both males and females (1). Factors contributing to the onset and progression of NAFLD include diet, lifestyle, genetics, gender, ethnicity and genetic polymorphisms. Since there are no FDA-approved treatment strategies for NAFLD, current treatment strategies focus on treating the comorbidities associated with NAFLD, such as T2DM, insulin resistance, obesity and hypertension (1, 4, 10).

Diet plays a major role in the onset and progression of NAFLD; and the diet most commonly associated with NAFLD is the western diet (WD) (11–16). The WD is a diet high in saturated, monounsaturated and trans-fat, cholesterol, simple sugar, low in fiber, and essential fatty acids, i.e., ω3 and ω6 polyunsaturated fatty acids (PUFA). While clinical and histological features of NAFLD are well-defined, the role of chronic ingestion of a WD on the pathogenesis of NAFLD and its progression to NASH is not fully elucidated. Particularly relevant is the lack of a comprehensive understanding of the early effects of an unhealthy diet on the onset and progression to NASH. This further encumbers the identification of biomarkers that may be useful in the diagnosis and treatment of NALFD before the onset of hepatic injury leading to fibrosis, cirrhosis, HCC and liver failure. Most time-course studies using preclinical mouse models designed to assess liver status in response to diet have the first time point after 8 weeks on a WD (17, 18). It is reasonable to expect there are earlier events leading to changes in liver function that set the stage for NASH.

Our aim is to identify early changes in the hepatic transcriptome linked to NASH pathogenesis that occur in a preclinical mouse model of NASH in response to the WD. Accordingly, we used an established mouse model for diet-induced NASH, i.e., the low-density lipoprotein receptor knockout (*Ldlr^−/−^*) mouse, fed a commercially available western diet (WD) (19–24). *Ldlr^−/−^* mice fed the WD become obese and develop markers of insulin resistance, i.e., elevated HOMA-IR, dyslipidemia, endotoxinemia and elevated markers of hepatic macrosteatosis, inflammation, fibrosis and hepatic injury [alanine aminotransferase (ALT) and aspartate aminotransferase (AST)] (19, 21, 25). Herein, we report that 1 week on the WD is sufficient to induce multiple hepatic markers of NASH that precede liver injury as manifested by hepatic macrosteatosis and fibrosis. These early changes in the hepatic transcriptome likely set the stage for disease progression resulting in significant liver injury and NASH.

## Materials and methods

### Animals and diets

All procedures for the use and care of animals used in our laboratory research were followed and approved by the Institutional Animal Care and Use Committee at Oregon State University (OSU). The study described below used two-month-old female and male *Ldlr^−/−^* [B6;129S7-Ldlr*^Tm1Her^*/J mice, stock# 002207] purchased from Jackson Laboratories. The study was carried out concurrently with both female and male mice. Mice were housed (5 mice/cage) at the OSU Linus Pauling Science Center vivarium in the same room and handled by the same personnel throughout the study. Mice were maintained on a 12-h light/dark cycle.

Mice were fed a purified low-fat diet (LFD) [Research Diets: D12450K] for 2 weeks prior to initiating the feeding trial to acclimate the mice to a purified diet and the vivarium. The 40 weeks’ time-course study of female and male mice consisted of two randomized groups for each sex: the LFD [Research Diets: D12450K] group and the Western Diet (WD) [Research Diets: D12079B] group. The purified LFD contained 20% of energy as protein (casein, cysteine), 70% energy as carbohydrate [corn starch (52%), maltodextrin (14%), sucrose (0.4%)], 10% energy as fat (soybean oil, lard) and cholesterol (0.002 mg/g) of diet. The purified WD contained 17% energy as protein (casein, methionine), 43% energy as carbohydrate [sucrose (30%); corn starch (10%), maltodextrin (3%)], 40% energy as fat (butter, corn oil), and cholesterol at 1.5 mg/g of diet. Both diets contained a vitamin and mineral mix and fiber, while the WD contained an additional antioxidant. The energy density of the LFD and WD was 3.82 kcal/gram and 4.67 kcal/gram, respectively.

**Figure 1:**
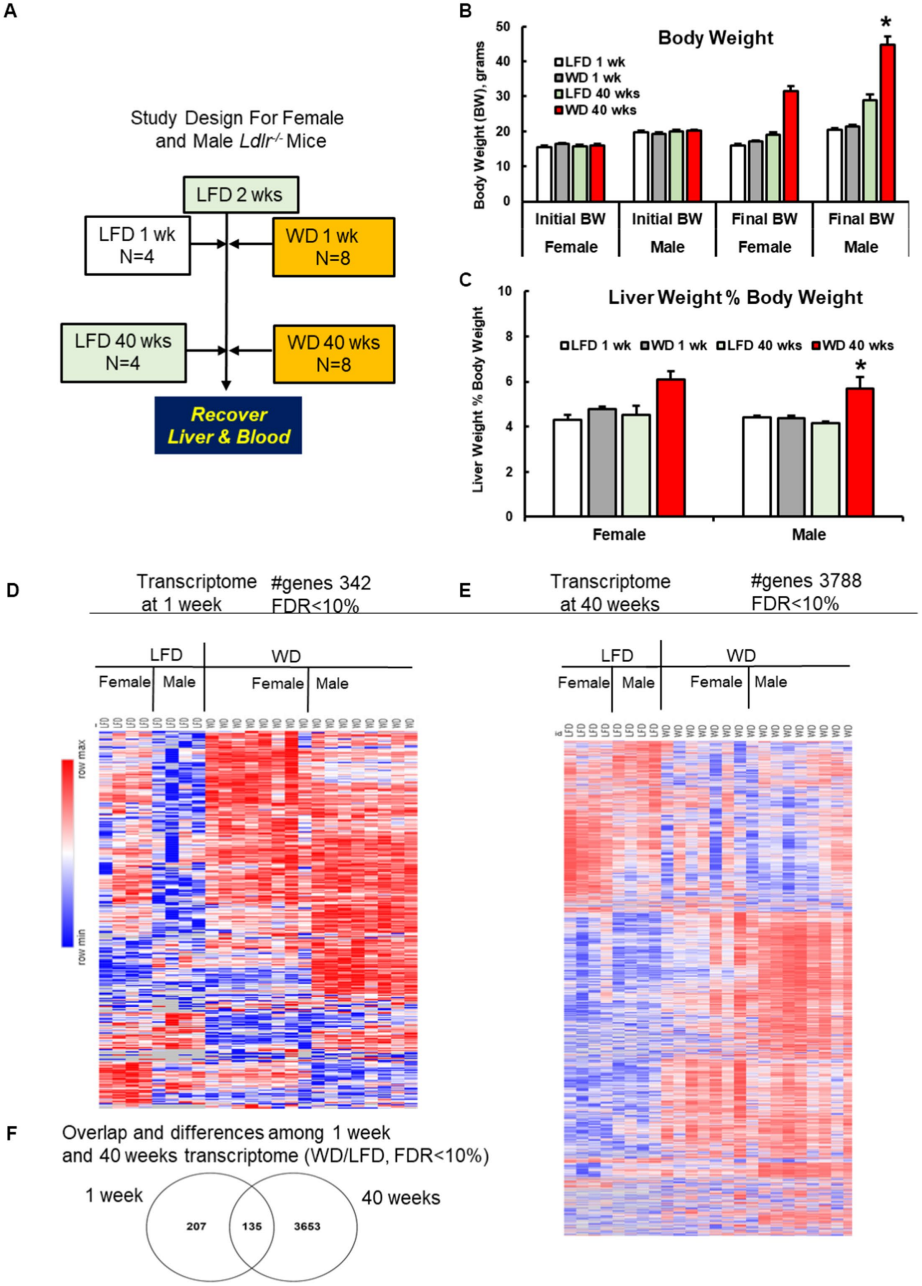
Study design, anthropometric features and hepatic transcriptome. **(A)** Study design for female and male *Ldlr*^−/−^ mice fed a low-fat diet (LFD) or western diet (WD) for 1 and 40 weeks. **(B,C)** WD effects on anthropometric markers, including **(B)** body weight (BW) of female and male mice after 1 and 40 weeks on the LFD and WD. Bar plot represents the mean +/− stderr and using Tukey’s post-hoc test for significance (*). **(C)** Liver weight as a % of body weight (LW%BW). LW%BW is a measure of hepatomegaly, a feature associated with fatty liver disease. **(D,E)** Heatmap of the hepatic transcriptome analysis of the female and male *Ldlr*^−/−^ mice after 1 week **(D)** and 40 weeks **(E)** on the LFD and WD. The heat map was prepared using the geometric mean of gene expression of LFD and WD fed female and male mice. Results are represented as differential gene expression between WD, and LFD-fed mice with an FDR < 10%. **(F)** The overlap and differences among early and late transcriptome from LFD and WD-fed mice shown in panels **(A,B)**. Hepatic transcripts increased or decreased by the WD are described in Supplementary Figures 2A–F.

After 2 weeks on the LFD, female and male mice were maintained on the LFD for 1 and 40 weeks (4 mice/gender and time point), while the remaining female and male mice were switched to the WD (8 mice/gender and time point; Figure 1A). Mice were weighed and had their health assessed twice weekly. Chow remaining from the previous feeding was weighed, discarded and fresh food added. Mice were euthanized by CO_2_ at 1 and 40 weeks on the WD; and 1 and 40 weeks on the LFD. Upon sacrifice, liver and blood was collected and processed for analyses as described previously (23, 24).

### Liver histology

Mouse livers (~100 mg) were fixed in buffered formalin, paraffin embedded, sliced and stained with hematoxylin–eosin (H&E) or Pico Sirius red (PCR; Nationwide Histology, Veradale, WA) (22, 24). Each slide contained 2 to 4 liver slices. Images were photographed using an Olympus inverted microscope equipped with a digital camera. Liver histology was scored by board-certified veterinary pathologists for hepatic macrosteatosis and fibrosis.

### RNA extraction and RNA sequencing library preparation

Liver RNA was extracted using Trizol (Life Technologies) (20, 21, 24), quantified and used for RNA sequencing (RNASeq) analysis following methods previous described (26). Briefly, RNA was quantified, and cDNA was prepared using the qScript reverse transcription kit from Quantabio (Thermo-Fisher). The qPCR procedure used Perfecta SYBR mix (Quantabio) and StepOne Plus RTPCR and software (Applied Biosystems). The RNA libraries were prepared using Quant 3’ mRNA-Seq Library Prep Kit (Lexogen) and sequenced using Illumina NexSeq. Sequences were processed to remove adapter, poly A and low-quality bases by BBTools^1^ using the bbduk parameters: k = 13, ktrim = r, fortrimleft = 12, useshortkmers = t, mink = 5 qtrim = r, trimq = 15, minlength = 20. Reads were aligned to the mouse genome and transcriptome (ENSEMBL NCBIM37) using Tophat (v2.1.1) (27, 28). The number of reads per million for mouse liver genes were counted using HTSeq (v 0.6.0) and quantile normalized. BRB-ArrayTools was used to identify differentially expressed genes between treatments.

### RNA extraction and quantitative RT-PCR

Total RNA was extracted from mouse liver, and specific transcripts were quantified by quantitative RT-PCR (qRT-PCR). Primers for each transcript are listed in Supplementary Table 5. Cyclophilin was used as the internal control for all transcripts as described in the references (22, 24).

### Heatmap, clustering, and summation of gene expression per cluster

Pair-wise comparison of genes between all 4 groups among 1 week (LFD and WD) and 40 weeks (LFD and WD) with established interaction between time and diet (FDR < 0.1, two-way ANOVA) were selected for downstream analysis. The k-mean clustering for geometric mean expression from the individual time points and diet were performed using Morpheus,^2^ a heatmap was created, genes belonging to each cluster were identified. The geometric mean expression for each gene in the cluster was summarized and a single value was obtained as representative for the cluster at each time point and diet.

### Single-cell RNA sequence data reanalysis

We used the single cell dataset (GSE129516) that was obtained from single cell RNA-sequence on non-parenchymal cells of healthy and NASH mouse livers (29). We reanalyzed the dataset and used it for assignment of cell type for the genes in our bulk RNASeq data. The standard method was followed as described previously for the analysis, filtering, normalization, identification of clusters and cell types.

### Single-cell RNA sequence for assignment of gene to a specific cell type

Briefly, the normalized unique molecular identifier or UMI > 1.0, with a significant fold change and uniquely expressed genes in a cell specific cluster were then assigned to that specific set of genes in the time course bulk RNASeq data to indicate they belong to that specific cell type (30).

### Gene ontology and functional enrichment

Functional enrichment of clusters was then performed using Metascape with mouse genome as background (31).

### Statistical analysis

Statistical analysis of mouse anthropometric features and histology used the MS-Excel statistical package for determining the mean and standard deviation. Mouse transcriptome data are expressed as geometric means of replicates. Group comparisons were performed using an unpaired *t-test* and two-way analysis of variance (ANOVA), followed by multiple comparisons tests, with *p* < 0.05 indicating statistical significance. A false discovery rate (FDR) value of 0.1was considered highly significant. Details of statistical analyses are described in the corresponding figure legends. The GraphPad Prism 9.0, BRB-ArrayTools^3^ and R statistical packages were used for the transcriptome and single cell data analysis.

## Results

Female and male mice were fed the LFD and WD for 1 week and 40 weeks. One week of WD feeding had no significant effect on body weight or liver weight as a percentage of body weight (LW%BW; Figures 1B,C). LW%BW is an indicator of hepatomegaly and a marker of liver disease (19). After 40 weeks on the diet, however, both body weight and LW%BW were significantly increased in WD-fed mice when compared to mice fed the LFD for 40 weeks. The effect of the WD was seen in both female and male mice.

We next examined hepatic histology for evidence of liver disease. Mice fed the LFD for 1 and 40 weeks and the WD for 1 week revealed no histological evidence of liver disease, such as steatosis or fibrosis. After 40 weeks on the WD, however, both female and male mice displayed histopathological evidence of NASH, which included both hepatic macrosteatosis and fibrosis (Supplementary Figures 1A,B). Histological scoring indicated that only mice fed the WD for 40 weeks displayed features of NASH. These results are consistent with our previous studies on WD-induced NASH in female and male mice (21, 24).

We next examined the hepatic transcriptome (available at NCBI-National Center for Biotechnology Information, accession number GSE223193) obtained from the 8 groups of mice: female and male mice fed the LFD and WD for 1 and 40 weeks. The RNASeq approach identified 10,162 transcripts in each of the 8 groups. We identified hepatic transcripts that were significantly affected after mice were fed the WD for 1 and 40 weeks. Our criterion for significance is: ≥2-fold increase or ≤ 0.5-fold decline in abundance, i.e., WD/LFD with an FDR ≤ 0.1. The heat maps reveal diet and gender effects on the hepatic transcriptome in mice fed the LFD and WD for 1 and 40 weeks (Figures 1D,E). This report, however, focuses on WD effects on the hepatic transcriptome.

Since we were interested in identifying WD-regulated transcripts common to both female and male mice, we pooled the RNASeq results of female and male mice. Accordingly, after 1 week on the WD, 342 transcripts were significantly different between LFD and WD-fed mice, which represents ~3.4% of the hepatic transcriptome (Supplementary Table 1). Of these, 270 transcripts were significantly increased while 72 transcripts were significantly decreased by WD feeding (Figure 1D; Supplementary Figures 2A,B). After 40 weeks on the WD, 3,788 transcripts were significantly affected by the diet, representing ~37% of the hepatic transcriptome (Supplementary Table 2). Of these 2,385 were significantly increased, while 1,403 were significantly decreased by WD feeding (Figure 1E). Of particular interest were transcripts that were affected by the WD early, after 1 week, and persisted to 40 weeks on the WD. Our analysis identified 135 transcripts responding to the WD after 1 week that were also altered by the WD after 40 weeks (Figure 1F).

Further gene enrichment analysis was performed for the up-and down-regulated transcripts in the 1 week and 40 weeks gene sets (Supplementary Figures 2A–E). Interestingly, the early onset changes reveal a path toward induction of an acute phase response, several dysregulated metabolic pathways and induction of inflammatory markers characteristic of NASH (Supplementary Figures 2C,D). While not all changes occurring in the liver transcriptome in response to the WD are related to NASH pathology, many of these genes are indicative of the dysregulated metabolic (*Lgals1, Fabp5, Lpl and Hk2*) and inflammatory processes (*Itgax, Cd68, Sparc* and *Cyba*).

### The transcriptome analysis for common genes with interactions of time and diet reveal early gene markers of NASH

**Figure 2:**
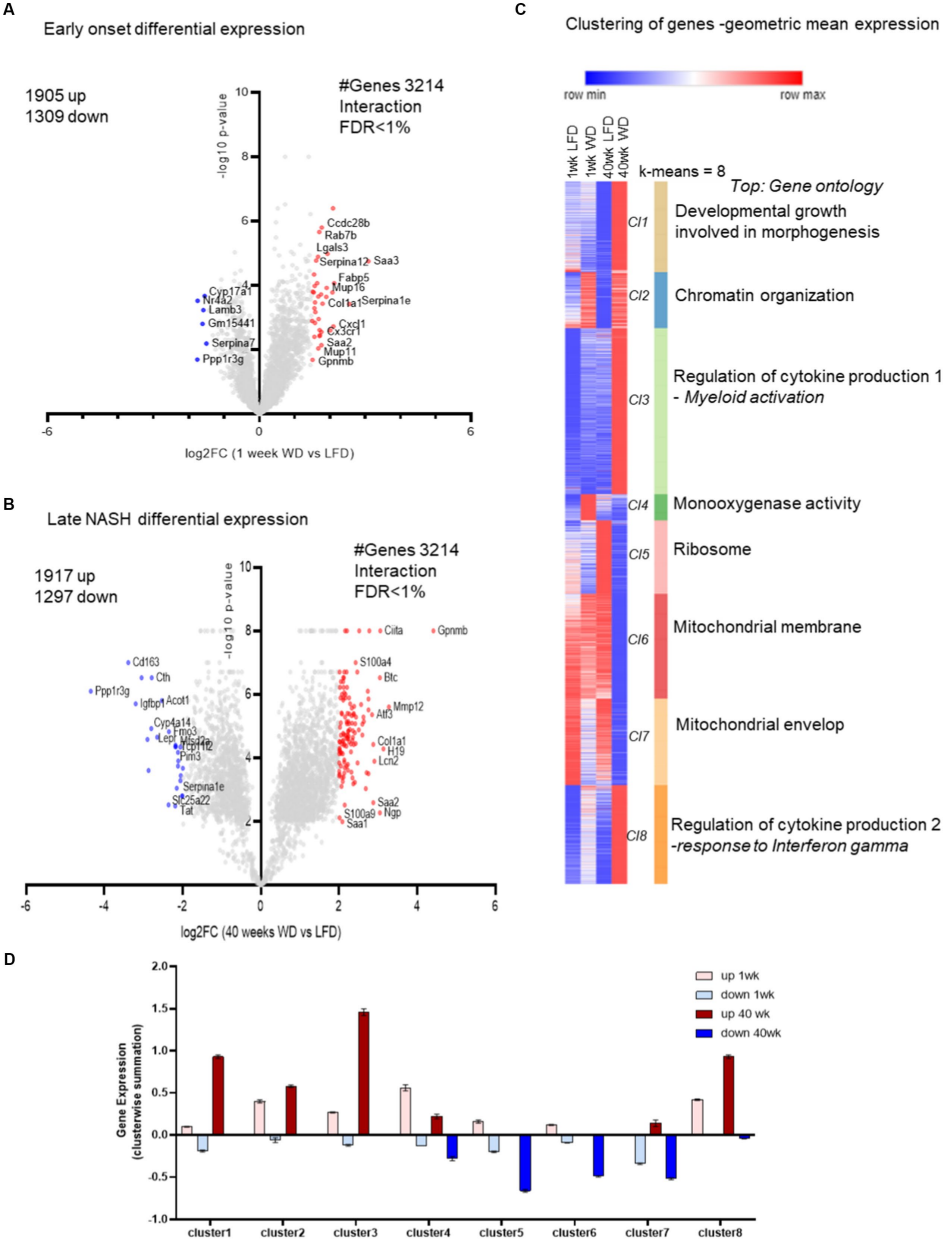
Pair-wise comparison between all groups: 1 and 40 weeks and with time and diet interaction (FDR < 0.1). In this analysis, we pooled the transcriptomic data of female and male mice for the time and diet comparison. Specifically, a time (1 vs. 40 weeks) and diet (LFD vs. WD) interaction (two-way ANOVA) with an FDR < 0.1. **(A)** Time and diet interactions, early 1 week log2 fold change (FC; WD/LFD) from the gene expression with genes marked in red to indicate induction and blue to indicate repression, with y-axis corresponding to *p*-values indicated in-log10 scale. **(B)** Similar volcano plot for the hepatic transcriptome at 40 weeks with genes marked in red to indicate induction and blue to indicate repression, with y-axis corresponding to *p*-values indicated in-log10 scale. **(C)** The heatmap from clustering of all 4 groups of hepatic transcriptomes for time and diet i.e.,1 week and 40 weeks and LFD and WD. The eight clusters (*Cl1-Cl8*) formed with k-mean clustering also shows top functional enrichments for the individual clusters. The color indicates red for row max and blue for row min gene geometric mean expressions. **(D)** The bar plot is a summarized median gene expression value from clusters for each dataset from the heatmap (as detailed in Materials and methods).

We next analyzed and compared the early onset changes in the hepatic transcriptome at 1 week that are associated with NASH at 40 weeks in a diet and time-dependent manner (Figures 2A,B). We identified 3,214 transcripts that were regulated by the WD after both 1 and 40 weeks (with a two-way ANOVA for merged set of genes from 1 week and 40 weeks, diet and time interaction FDR < 1%; Supplementary Table 3). These common genes are associated with NASH pathogenesis, and include proteins linked to hepatic fibrosis (collagen 1a1; *Col1a1, Col1a2 and Col3a1*), metabolic syndrome [MetS; galectin 3 (*Lgals3*), lipoprotein lipase (*Lpl*), glycoprotein nmb (*Gpnmb*), fatty acid binding protein-5 (*Fabp5*), and the acute phase response (*Saa1*-3)]. All of these transcripts were induced in response to WD feeding. In addition to playing a role in hepatic fibrosis, many of these proteins are reported to promote chemotaxis, cytokine induction, matrix metalloprotease-9 (Mmp9) release, the generation of reactive oxygen species [ROS (genes as in 40 weeks, Ecto-NOX Disulfide-Thiol Exchanger 2, *Enox2*)], and macrophage differentiation in response to inflammation and tissue injury (Figures 2A,B) (32).

After 40 weeks on the WD, additional transcripts associated with NASH were induced that were not induced after 1 week on the WD. These transcripts included, matrix metalloprotein-12 (*Mmp12*), betacellulin (*Btc*), activating transcription factor 3 (*Atf3*) and S100 calcium binding protein A9 (*S100a9*). Recent studies suggest these late transcripts play a major role in the pathology of MetS, type 2 diabetes (T2D) and NASH (33).

Among the significantly down-regulated genes, acyl CoA thioesterase 1 (*Acot1*) expression is regulated by the fatty acid-regulated nuclear receptor peroxisome proliferator activated receptor (PPARα) (24). *Acot1* affects fatty acyl CoA levels and protects the liver from excess FA oxidation and the ensuing oxidative stress and inflammation (34). Flavin-containing monooxygenases (*Fmo3*), an enzyme that is involved in metabolism of dietary and xenobiotic compounds, was significantly down-regulated by the WD. Some studies suggest *Fmo3* plays a role in metabolic diseases including diabetes (Figure 2B) (35).

### Gene ontology for the common genes from early to late transcriptome analysis suggests increased cytokine production and compromised mitochondrial function

Herein we reveal the cluster-wise gene ontology of the transcripts significantly regulated by the interaction of diet and time. K-mean clustering identified 8 distinct clusters, i.e., C1–C8 (Figure 2C). The top enrichment categories for the individual clusters were, Developmental Growth involved in morphogenesis, Chromatin Organization, Regulation of Cytokine Production (with myeloid activation), Monooxygenase Activity, Ribosome, Mitochondrial Membrane and Mitochondrial Envelope. There were two distinct clusters enriched with inflammatory pathways and two clusters enriched with mitochondrial organization indicating the importance of these responses of the hepatic transcriptome to WD feeding. The distinction between the mitochondrial clusters is evident in terms of functional enrichment. The Mitochondrial Membrane cluster is also enriched with mitochondrial matrix and tricarboxylic acid cycle (TCA) processes, while the Mitochondrial Envelop cluster is additionally enriched with fatty acid metabolism and cholesterol metabolic processes. The cluster of down-regulated genes enriched in structural constituents of Ribosomal function and translation are consistent with our previous analysis with WD (33). Clusters C1-C3 and C8 (Figure 2C) had transcripts highly upregulated after 40 weeks of WD feeding. Importantly, the transcripts down regulated after 40 weeks on the WD were distributed among clusters C4–C7 (Figure 2D).

Clusters C2–C4 and 8 (Figure 2C) were the early indicators of WD induced gene functions such as monooxygenase activity (which is strikingly up regulated early on but down-regulated after 40 weeks on the WD; and included genes such as *Cyp2f2, Cyp2c50, Cyp2c23, Cyp2c54, Pah*, and *Akr1c12*), inflammatory cytokine production, myeloid activation, response to interferon-γ and growth. The clusters C4 and C6 (Figure 2D) had several biological processes down-regulated at 40 weeks, which included enrichment in lipid catabolic processes.

**Figure 3:**
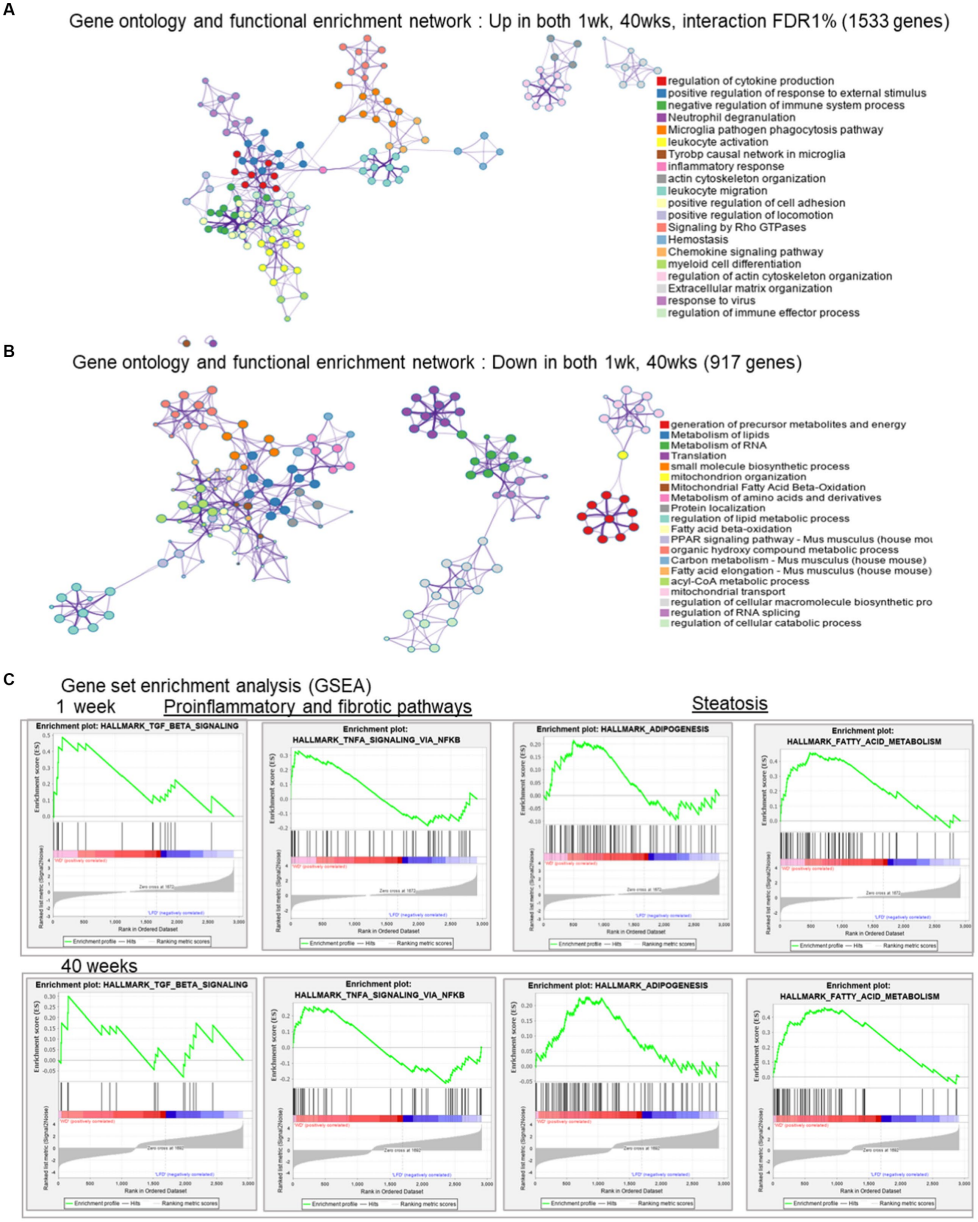
Gene ontology and functional enrichment networks. **(A)** Gene ontology and function al enrichment network: in both 1 and 40 weeks, interaction FDR < 0.1; 1,533 genes. **(B)** Gene ontology and functional enrichment: Down regulated in both 1 week, 40 weeks, for genes with diet and time interaction FDR < 0.1; 917 genes. **(C)** Gene set enrichment analysis (GSEA) from the gene expression at 1 week (LFD and WD) and 40 weeks (LFD and WD), respectively, against the hallmark pathways for selected representative processes.

WD-induced transcripts (1,533, expressed positively with FDR < 1%) at both time points were enriched for cytokine production and leukocyte activation indicating a proinflammatory process (Figure 3A). These interacting networks of biological processes show the connections between the major components of immune system, such as leukocyte activation, positive regulation of response to external stimulus and leukocyte migration. In contrast, the 917 transcripts suppressed by the WD (expressed negatively with FDR < 1%) were associated with pathways involved in lipid and RNA metabolism, as well as mitochondrial fatty acid β-oxidation. This outcome suggests the negative impact of the WD on hepatic energy metabolism and mitochondrion organization (Figure 3B). Specifically, these interacting networks of biological processes are indicative of the earliest events in NASH pathogenesis.

To assess pathway enrichment by WD at early and late timepoints, we used Gene Set Enrichment Analysis (GSEA) (36) with these common genes across both time points (FDR < 0.1). Surprisingly, we could see early indicators of steatosis, inflammation and fibrosis pathway enrichment at 1 week and more so after 40 weeks. Specifically, TGFβ and TNFα signaling were increased via the NFκβ pathway indicating progression of profibrotic and inflammation pathways. The hallmarks of adipogenesis and fatty acid metabolism enrichment are likely early indicators of steatosis (Figure 3C).

### Liver cell-specific gene assignments indicate macrophage and other specialized liver cells respond rapidly to the WD

Information regarding the association of specific transcripts with specific hepatic cell types provides a better understanding of how the WD effects liver cellular function. Accordingly, we have matched specific transcripts with a specific hepatic cell types using the publicly available cell-specific RNASeq data base for NASH in C57BL/6 J mice (GSE129516) (29). The inferred cell types for our cell-free bulk RNA sequence from both 1 week and 40 weeks on the WD gave us an opportunity to unravel functional and pathological processes to specific liver cells from this early and late hepatic transcriptomic data set.

**Figure 4:**
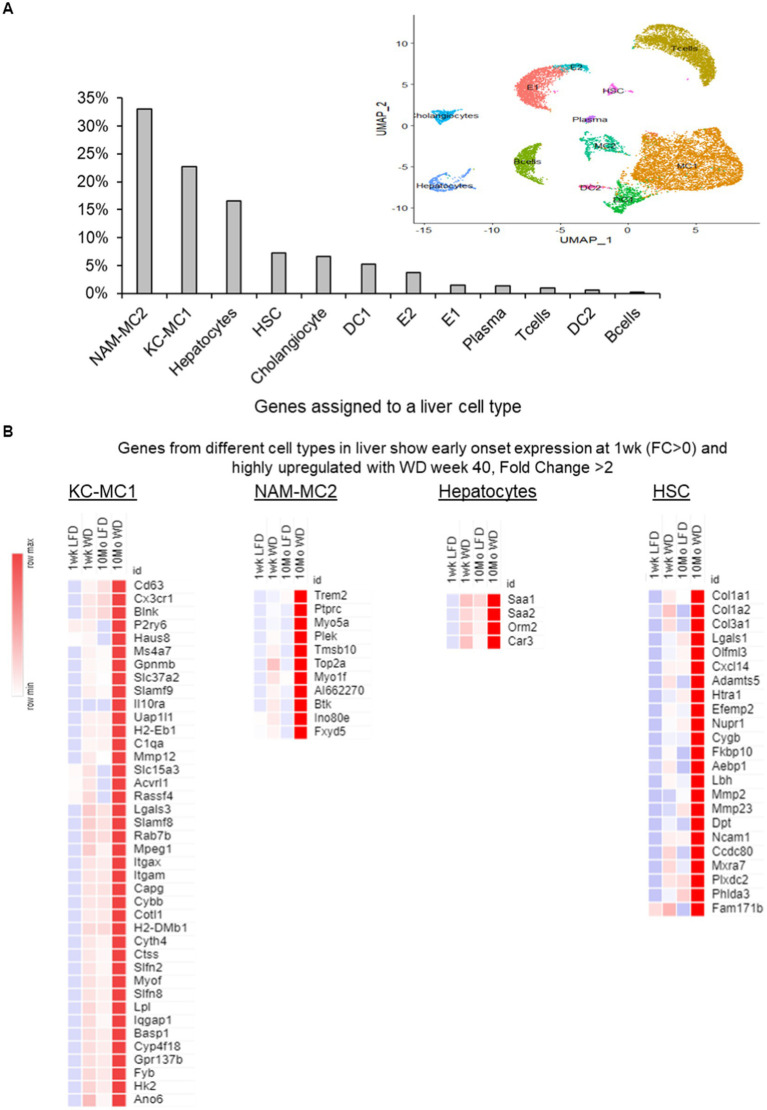
Genes assigned to different liver cells with a 1 week and 40 weeks transcriptome interaction (FDR < 0.1) using information from reanalysis of the NASH single cell RNASeq analysis (GSE129516). **(A)** A bar plot of the percentage of genes assigned to different cells in liver from the reanalysis of NASH mouse single cell RNA sequence (GSE129516) in a pair-wise comparison between all 4 groups of the transcriptome with interaction FDR < 0.1. **(B)** The most prominent cell types from the cell assignments with a subset of specific genes from different cell types in liver show early onset expression at 1 week (fold change; FC > 0) and are highly upregulated with WD at week 40 (fold change; FC > 2). KC-MC2, Kupffer cell-macrophage; NAM-MC2, Nash-associated macrophage; HSC, hepatic stellate cells; DC, dendritic cells, E, endothelial cells.

Broadly, 12 different cell types in liver (~15,260 single cells from mouse NASH dataset) that could be identified distinctly from the reanalysis of the single cell transcriptome could be assigned to genes in our hepatic transcriptome analysis. The highest number of genes were assigned to macrophages, more than 50% of all genes (1,374/3,214 genes). The number of genes associated with liver-specific and functionally specialized hepatocytes (~15% of the gene assigned), hepatic stellate cells (HSC) and cholangiocytes (<10% each) were lower (Figure 4A). There was a similar trend in cell assignments observed during the assignment of genes individually to 1 week and 40 weeks of WD-transcriptome data sets (Supplementary Figures 3A,B).

Interestingly, the macrophage subtypes, such as Kupfer like cells (KC-MC1) and NASH-associated macrophages (NAM-MC2), distinctly over-express many marker genes in the early and late time points. The KC-MC1 cells were identified with the classical Kupffer cell markers *Clec4f* and *Cd5l* and the NAM-MC2 were identified with *Trem2* higher expression. It is noteworthy that the KC-MC1 (cells with *Mmp12*^high^, *Trem2*^low^) were more enriched with proinflammatory genes and with genes associated with insulin resistance and metabolic inflammation (29, 33, 37). In the NAM-MC2 (cells with *Trem2*^high^, *Mmp12*^low^) marked increase in *Trem2* expression was observed at 40 weeks (Figure 4B). To identify the distinct sub-clusters in macrophages and to identify the early changes in the genes of a specific sub-cluster, we re-analyzed just the macrophages (38–41). The macrophages were grouped into four sub-clusters, the two major cell types described previously (KC-MC1 with about 2,517 cells and NAM-MC2 with about 1961 cells) along with two monocyte-derived macrophage subtypes (Ly6c2-hi cluster with about 1,377 cells and Ly6c2-low with about 348 cells) forming minor population of cells (Supplementary Figures 3C,D). Here the NAM-MC2 cells had a major part of the genes (~420 out of 487 genes) assigned with the interaction effects of diet and time. While the KC-MC1 has around 310 genes, Ly6c2-hi cells had 7 and Ly6c2-low had about 50 genes assigned among the interaction effects of diet and time, respectively (Supplementary Figure 3E). The induction of several genes in the NAM and KC-like macrophages at the early stage and continued over expression at 40 weeks indicate the highly active immune processes during the progression to NASH (Supplementary Figure 3E).

In the genes assigned to hepatocytes, the acute phase response genes (*Saa1, Saa2*, and *Orm2*) were markedly induced and overexpressed over the duration of 1 to 40 weeks feeding period of the WD indicating a modulation of metabolic and innate immune system activities (Figure 4B). In the hepatic stellate cell genes associated with genes associated with fibrosis (*Col1a1, Col1a2*, and *Col3a1*), cytokines/chemokines (*Cxcl14*) along with cell division (*Zfp281, Emp1, Dcn*) and activation markers (*Sparc, Col1a1, Gpx3*) were induced at early timepoints and significantly over-expressed after 40 weeks of WD feeding.

To evaluate the transcriptome (RNASeq) data studied, we independently verified the selected few important genes using qRTPCR to quantify transcript abundance and were chosen representatives of the differently regulated and from multiple cell types as described above (Supplementary Tables 4, 5). Evidently, they correlate significantly in both the approaches (qRTPCR vs. RNAseq) at each of the two timepoints (Supplementary Figures 3F,G) and with respective diets. Here, as the 40 weeks fed with WD represents the NASH disease state in mice and we have studies at similar (late) time points (19, 20, 24), this allows to us evaluate not only the technical issues (qRTPCR vs. RNAseq) but also to compare with similar gene profiles emerging from independent studies including the NASH single cell RNASeq analysis.

To investigate further, whether the differentially regulated genes that are indicative of pathogenic processes indeed have interactions, we took advantage of protein–protein interaction network. For ~150 of the top genes with expression at 1 week (fold change > 0) and those that are highly upregulated with WD at week 40 (fold change > 2, with interaction FDR < 0.1), the STRING mouse protein–protein interaction network (42) with medium interaction confidence of ~0.4 was investigated and analyzed. The clusters of interaction network thus obtained established that acute phase proteins, and proteins involved in oxidative stress, collagen formation and activated immune responses are altered in the early response to the WD (Supplementary Figure 4).

## Discussion and conclusion

The western diet is associated with multiple related diseases such as NAFLD/NASH, T2DM, cardiovascular disease (CVD) and MetS, commonly known as metabolic diseases (11, 14). Here we analyzed the rapid and long-term changes that occur after feeding female and male *Ldlr ^−/−^* mice a WD for 1 and 40 weeks. This is an established preclinical model of NAFLD/NASH that reveals all the hallmarks of chronic liver disease including hepatic macrosteatosis, inflammation, oxidative stress and fibrosis as well as other markers of metabolic disease such as obesity, dyslipidemia and insulin resistance (19, 21, 24).

In addition to RNASeq transcriptome analysis, we used multiple approaches for functional enrichment (43), gene set enrichment analysis, protein–protein interaction network, as well as single cell RNA sequence data available publicly to enrich the information regarding the cell-specific associations of the transcripts. This allowed us to discern the early onset activation of specific macrophage subtypes including NASH-associated macrophages, Kupffer cells and other specialized hepatic cells such as hepatocytes and stellate cells. These molecular events show the hepatic stellate fibrosis markers (*Col1a1, Col1a2*, and *Col3a1*), macrophage mediated cytokine production (*TGFβ* and *TNFα*), metabolic disease markers (*Mmp12*, *Trem2, Lgals3*, *Lpl*, *Gpnmb*, and *Fabp5*) and hepatocyte acute phase response (*Saa1, Saa2*, and *Orm2*) are transcriptional markers and cell signatures of the early response to the WD. This early onset model agrees with the previous literature (35, 36) on the processes of immune activation and dysregulated lipid and metabolic processes. Early monooxygenase activation and reduced ribosomal function are specific characteristics we have unearthed in this study that need to be further investigated in the future. Moreover, we have identified novel molecular targets that were previously unknown to be affected after such brief exposure to the WD (35, 36). This much-needed molecular and cell-associated detail are useful in directing early patient monitoring and therapeutic intervention.

Importantly, at the gene ontology and functional level with upregulated genes, inflammatory response and steroid metabolic process are the early onset functional processes associated with the onset of pathogenesis after 1 week of WD. While the downregulated processes included fatty acid metabolic process and regulation of lipid storage indicating dysregulation of hepatic metabolism at early stage of this progressive fatty liver disease.

The study described herein is the first study that examined early events in the process of WD induced NAFLD/NASH. The prevailing view of NASH onset and progression is that steatosis and lipo-toxicity precede inflammation and fibrosis. Our findings indicate otherwise, and these inflammatory changes occurred at a time preceding overt disease development, such as hepatosteatosis and fibrosis.

In summary, this study provides an in-depth analysis of early transcriptomic indicators of WD-mediated changes in the liver that persist throughout the 40 weeks WD feeding trial leading to NASH. The study includes an analysis of molecular, cellular, and functional interactions and biological networks associated with NASH. The identification of the early cell-specific markers may be useful in early clinical assessment and therapeutic intervention of NASH.

## Data availability statement

The datasets presented in this study can be found in online repositories. The names of the repository/repositories and accession number(s) can be found in the article/Supplementary material.

## Ethics statement

The animal study was reviewed and approved by Institutional Animal Care and Use Committee at Oregon State University (OSU).

## Author contributions

JP, AM, NS, and DJ: conceptualization, visualization, project administration, writing—original draft, and writing—review and editing. JP, MS, ZL, NN, KA, NS, AM, and DJ: methodology. MS, JP, CL, KA, NS, AM, and DJ: investigation. NS, DJ, and GT: funding acquisition. All authors contributed to the article and approved the submitted version.

## Funding

This work was supported by the National Institutes of Health grants R01 DK103761 (NS) and R01 DK112360 (DJ).

## Conflict of interest

The authors declare that the research was conducted in the absence of any commercial or financial relationships that could be construed as a potential conflict of interest.

## Publisher’s note

All claims expressed in this article are solely those of the authors and do not necessarily represent those of their affiliated organizations, or those of the publisher, the editors and the reviewers. Any product that may be evaluated in this article, or claim that may be made by its manufacturer, is not guaranteed or endorsed by the publisher.

## Footnotes

^1^https://doe.gov/data-and-tools/bbtools/

^2^https://software.broadinstitute.org/morpheus/

^3^https://brb.nci.nih.gov/BRB-ArrayTools/
