# Supplementary material for: Early transcriptome changes associated with western diet induced NASH in Ldlr−/− mice points to activation of hepatic macrophages and an acute phase response
Source: Front Nutr. 2023 Aug 7;10:1147602. doi: 10.3389/fnut.2023.1147602 (PMC10440380; doi:10.3389/fnut.2023.1147602)
Supplement: Supplementary file 2 [file Data_Sheet_1.pdf]

Early transcriptome changes associated with western diet induced NASH in *Ldlr*$^{-/-}$ mice points to activation of hepatic macrophages and an acute phase response.

Jyothi Padiadpu[3†*], Melinda H Spooner[1†], Zhipeng Li[2], Nolan Newman[3], Christiane V. Löhr[2], K. Denise Apperson[2&], Amiran Dzutsev[4], Giorgio Trinchieri[4], Natalia Shulzhenko[2], Andrey Morgun[3], Donald B. Jump[1*]

Supplementary Figures

Supplementary Figure 1

A

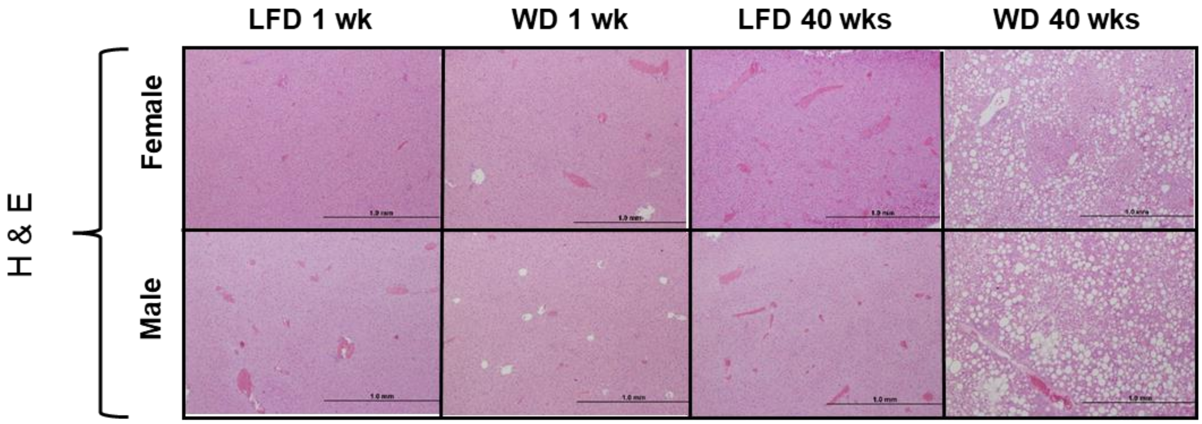

MAS

Female	0	0	0	1.1 +/- 0.4
Male	0	0	0	2.0 +/- 0.3

B

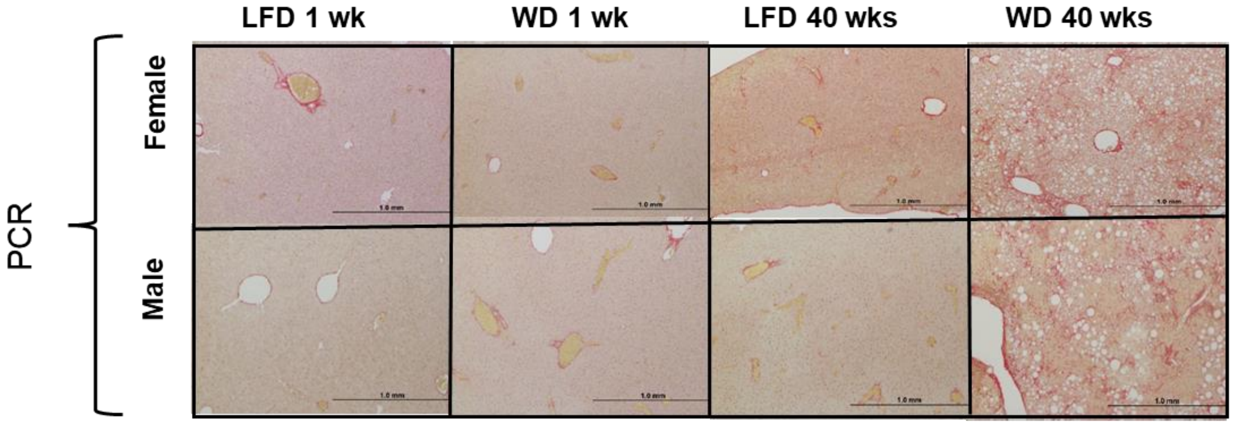

Fibrosis grade

Female	0	0	0	2.6 +/- 0.2
Male	0	0	0	2.1 +/- 0.4

Supplemental Figure 1. Representative hepatic histology of LFD and WD-fed mice after 1 and 40 wks on the two diets. **A.** Hepatic histology: Representative samples of liver prepared for histology as described in the Methods section. Liver samples were stained with A. hematoxylin and eosin (**H & E**) to reveal steatosis and **B.** Pico Sirius Red (**PCR**) to reveal collagen fibers, a marker of fibrosis (red). Scoring for both macrosteatosis (MAS) and fibrosis was performed by a board-certified veterinary pathologist.

Supplementary Figure 2

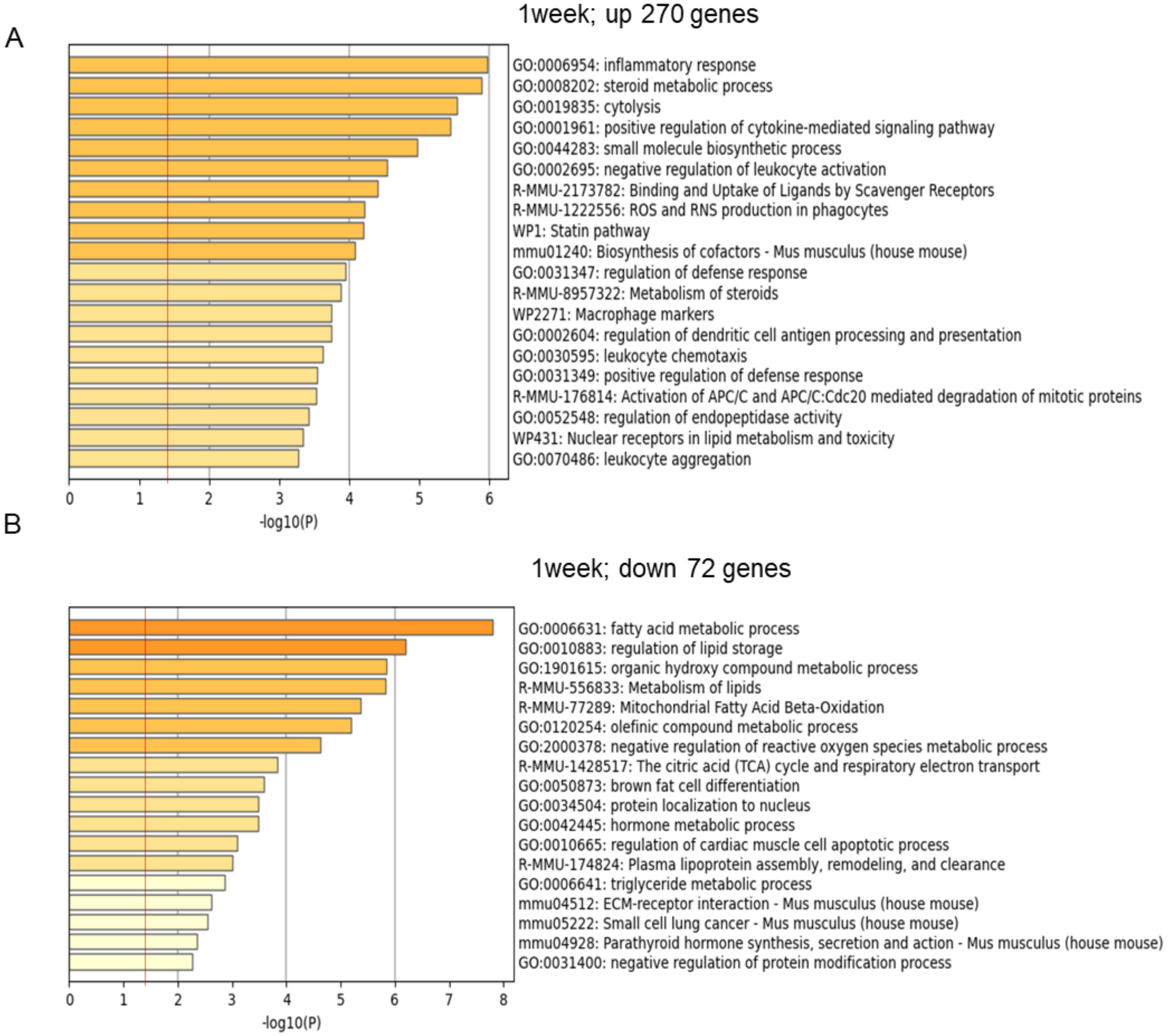

Supplementary Figure 2 *continued*

C

135 common genes in 1 week and 40 weeks

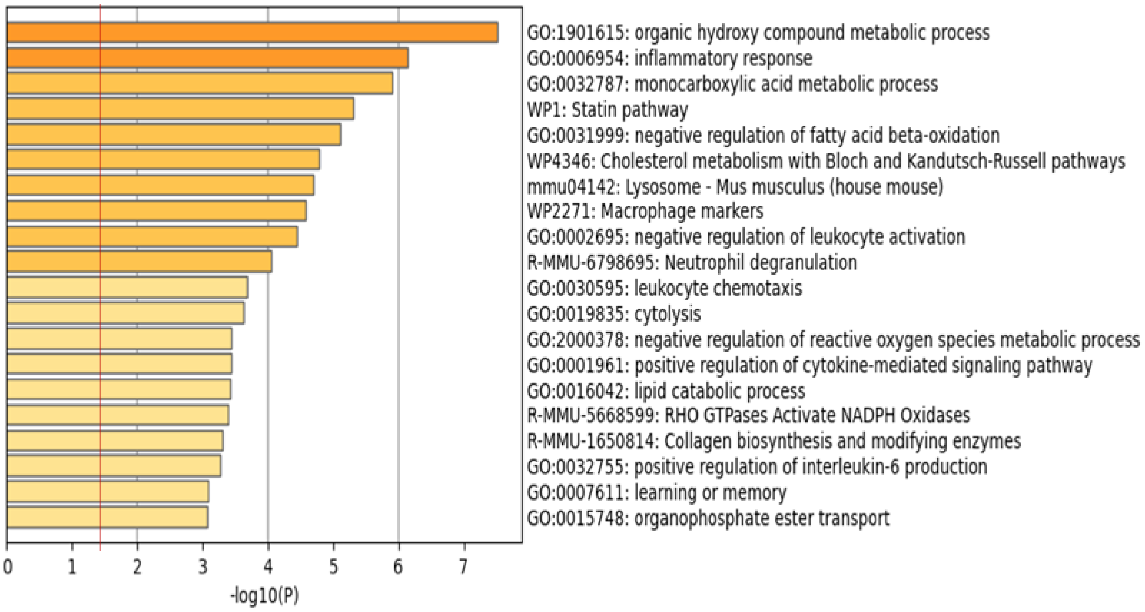

D

207 genes included exclusively in 1 week

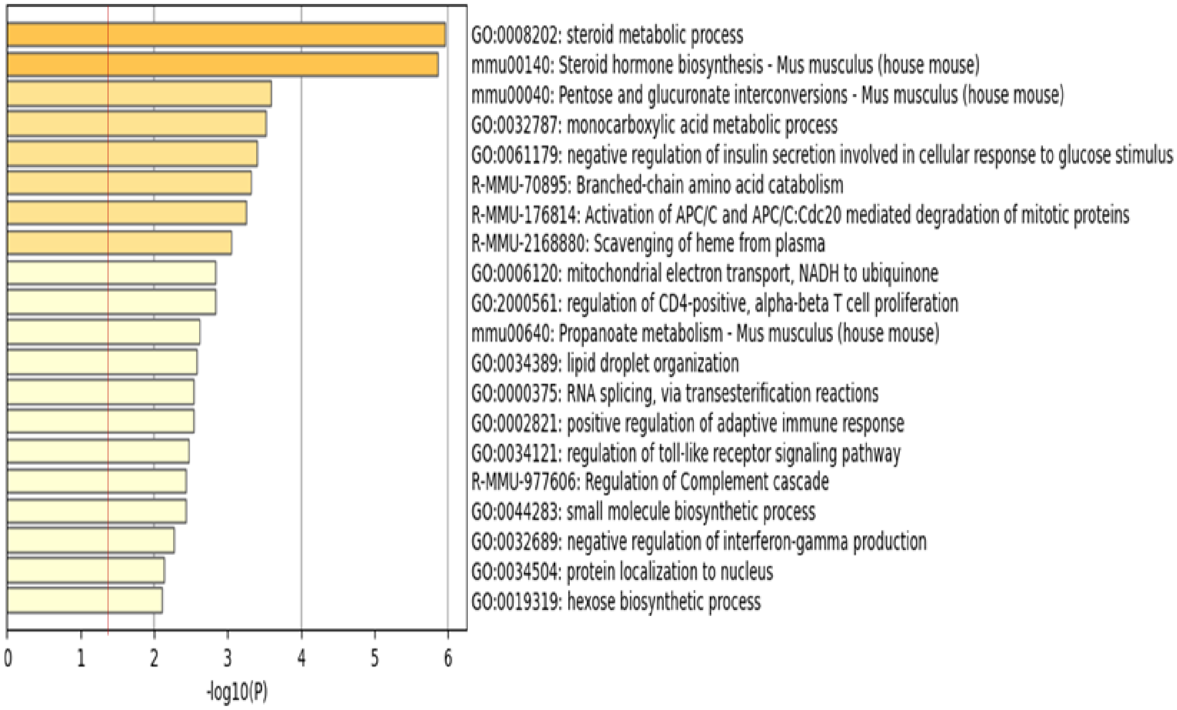

Supplementary Figure 2 *continued*

E

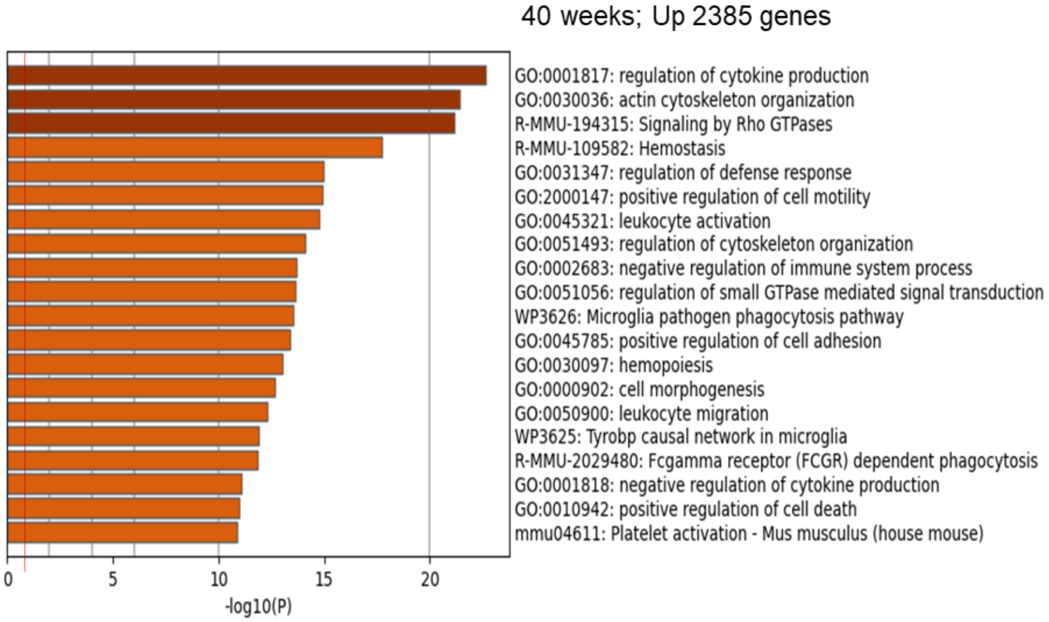

F

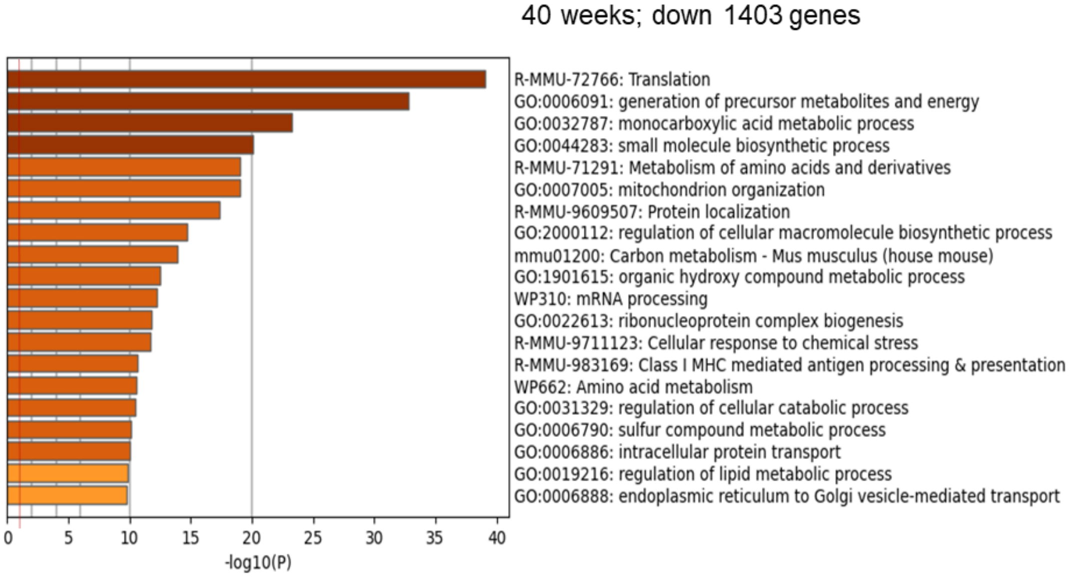

Supplemental Figure 2. **A**. The functional enrichment or gene ontology for the upregulated genes at 1 wk with indicating significance at and beyond a red dashed line, where data are displayed as -log10 (p-value). **B.** The functional enrichment or gene ontology for down-regulated genes at 1 wk, where data are displayed as -log10 (p-value). **C**. The common genes in 1

wk and 40 wks within the cutoff for FDR<10%. **D**. The unique set of genes at 1 wk within the cutoff for FDR<10%. **E.** The functional enrichment or gene ontology for the upregulated genes at 40 wks with indicating significance at and beyond a red dashed line, where data are displayed as -log10 (p-value). **F.** The functional enrichment or gene ontology for down regulated genes at 40 wks, where data are displayed as -log10 (p-value).

Supplementary Figure 3

No. of genes assigned to liver cell types based on single cell RNA seq at each of the 1 week and 40 weeks transcriptome (FDR<10%), WD to LFD comparisons

A

1 week

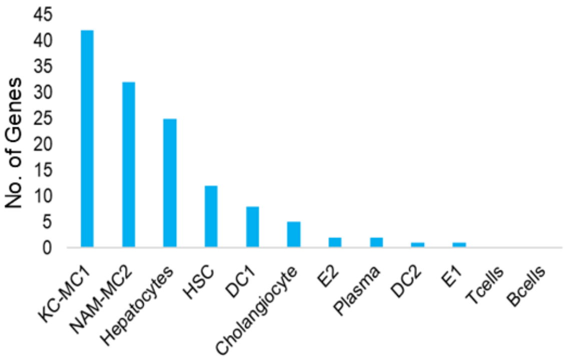

B

40 weeks

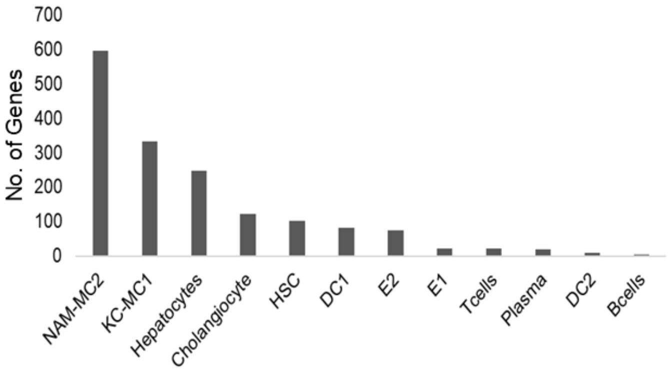

C Macrophage sub types

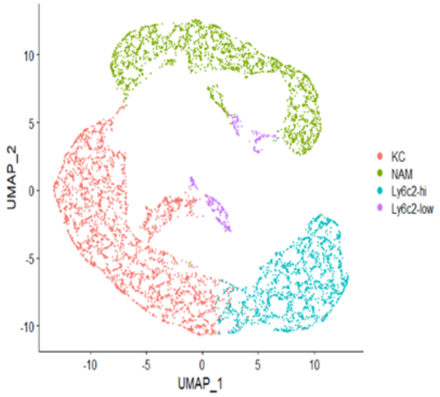

D Gene markers for macrophages and the sub types

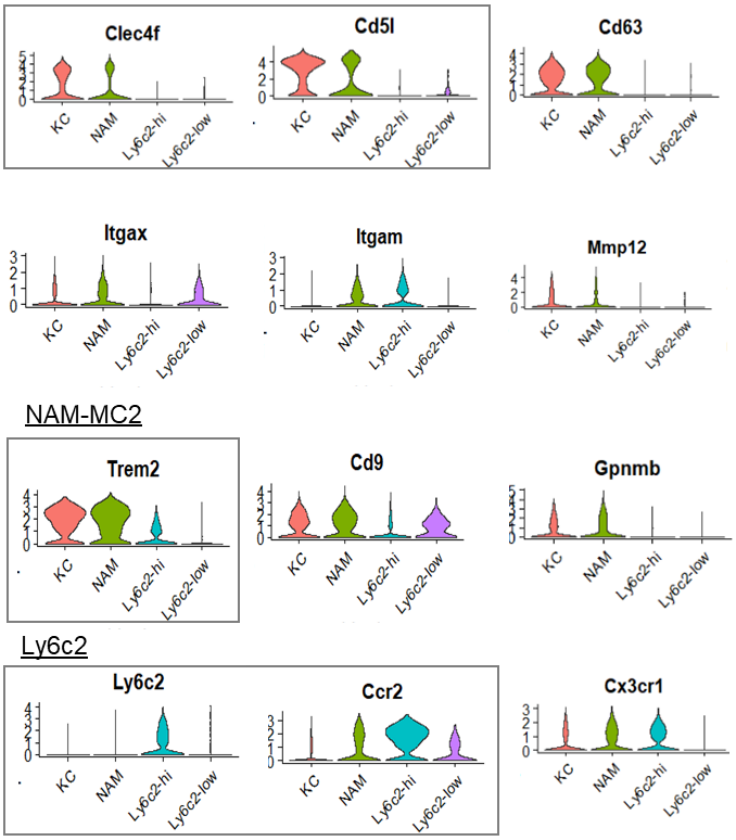

Genes with diet and time interaction FDR<1%

E

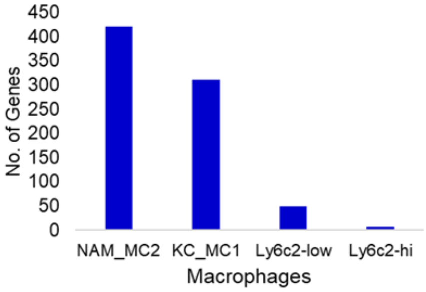

Supplementary Figure 3 *continued*

F

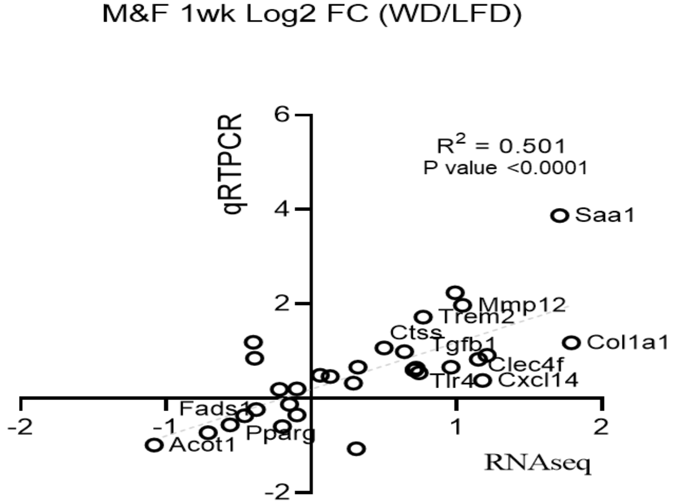

G

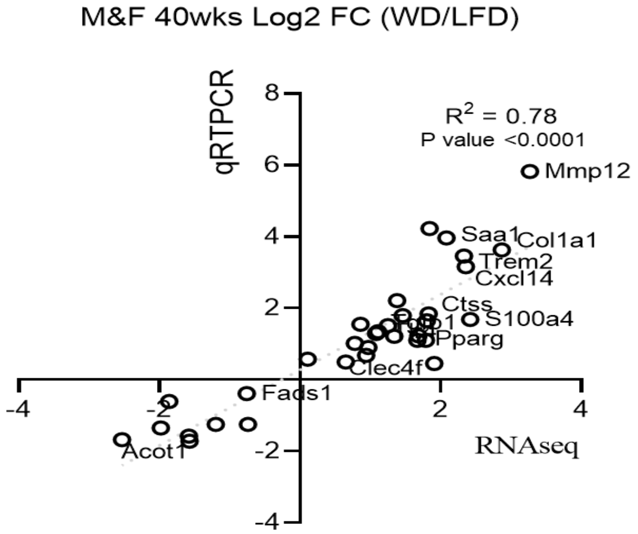

Supplemental Figure 3. A & B. No. of genes assigned to liver cell types based on single cell RNA seq to the transcriptome comparing WD and LFD at 1 wk and 40 wks time points (FDR<10%) respectively. **C.** The t-SNE plot for different macrophage subtypes identified in liver from the reanalysis of NASH mouse single cell RNA sequence (GSE129516). **D**. The gene markers to support the identified cell types based on literature. **E.** The macrophage subtypes and number of genes assigned to each of these cells in a pair-wise comparison between all 4 groups of the transcriptome with interaction FDR <1%. **F.** The XY plot for significant foldchange (log2 FC) for genes from the average of male and female (M&F) mice from the study between WD and LFD at 1 wk, showing qRTPCR vs RNAseq expression data with a corresponding R^2 and significance values. The labeled genes are the representative of upregulated, downregulated and cell type specific genes from the analysis **G.** Similar XY plot for significant foldchange (log2 FC) for genes from the average of male and female mice

from the study between WD and LFD at 40 wks, showing qRTPCR vs RNAseq expression data with a corresponding R^2 and significance values.

Supplementary Figure 4 Protein-protein interaction network for genes with expression at 1wk (FC>0) and highly upregulated with WD at week 40, Fold Change >2 with the interaction FDR <1%

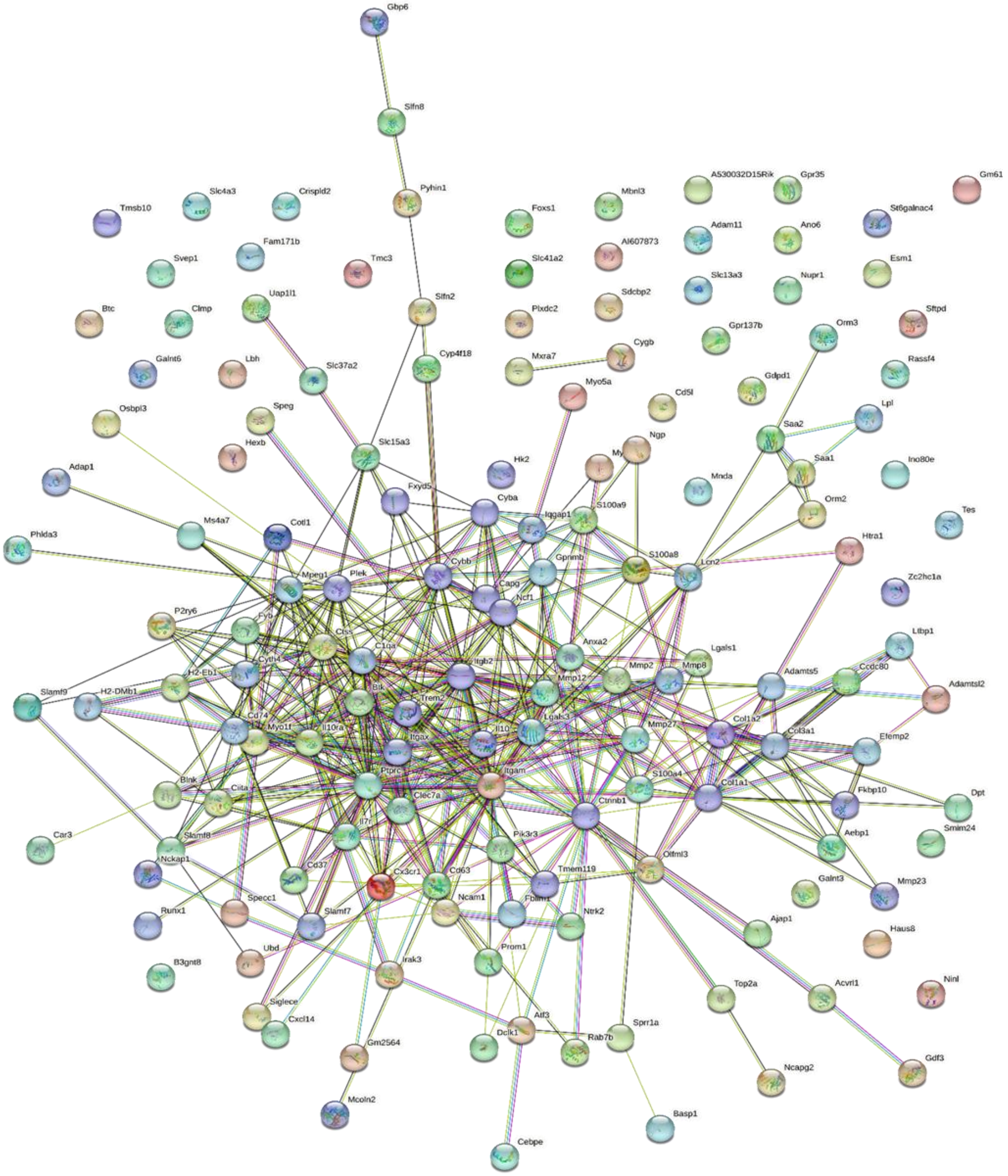

Network Stats

number of nodes:	149	expected number of edges:	81
number of edges:	352	PPI enrichment p-value:	< 1.0e-16
average node degree:	4.72		
avg. local clustering coefficient:	0.431		

your network has significantly more interactions than expected (what does that mean?)

Local network cluster (STRING)

cluster	description	count in network	strength	false discovery rate
CL:25269	Serum amyloid A protein, and Alpha-1-acid glycoprotein	3 of 5	1.93	0.0097
CL:17679	Superoxide-generating nad(p)h oxidase activity	3 of 6	1.85	0.0119
CL:14273	Banded collagen fibril, and peptidase m12b, adam-ts2	3 of 7	1.79	0.0142
CL:14056	Activated t cell proliferation, and cell-cell adhesion in respon...	3 of 10	1.63	0.0288
CL:17708	Mixed, incl. cathelicidin, and neutrophil aggregation	3 of 11	1.59	0.0322

Supplemental Figure 4. Protein-protein interaction (PPI) network derived for mouse genes from expression at both 1 wk (FC>0) and highly upregulated with WD at 40 wks, Fold Change >2 with the time and diet interaction FDR <1%. The PPI network stats and cluster description with FDR values are shown in the bottom legend.
